# Multi-Task Learning Based on Stochastic Configuration Networks

**DOI:** 10.3389/fbioe.2022.890132

**Published:** 2022-08-04

**Authors:** Xue-Mei Dong, Xudong Kong, Xiaoping Zhang

**Affiliations:** Collaborative Innovation Center of Statistical Data Engineering, Technology & Application, School of Statistics and Mathematics, Zhejiang Gongshang University, Hangzhou, China

**Keywords:** multi-task learning, neural networks, stochastic configuration, knowledge sharing and transfer, supervised mechanism

## Abstract

When the human brain learns multiple related or continuous tasks, it will produce knowledge sharing and transfer. Thus, fast and effective task learning can be realized. This idea leads to multi-task learning. The key of multi-task learning is to find the correlation between tasks and establish a fast and effective model based on these relationship information. This paper proposes a multi-task learning framework based on stochastic configuration networks. It organically combines the idea of the classical parameter sharing multi-task learning with that of constraint sharing configuration in stochastic configuration networks. Moreover, it provides an efficient multi-kernel function selection mechanism. The convergence of the proposed algorithm is proved theoretically. The experiment results on one simulation data set and four real life data sets verify the effectiveness of the proposed algorithm.

## 1 Introduction

In supervised machine learning, we often encounter situations that establishing models for several related tasks, such as searching cancer sites, identifying cancer types, judging cancer stages and so on, based on cancer image data. Generally, these tasks are undertaken separately, which we refer to as single-task supervised learning (STSL) in traditional machine learning (Ben-David and Schuller, 2003). These models do not consider the correlation among multiple tasks so some common information in model parameters or data features is lost. In particular, when the training sample size of a single task is insufficient, it is difficult for STSL to capture enough information, which results in poor generalization performance. Multi-task supervised learning (MTSL) provides a solution for such a situation. It improves the performance of each task by setting shared representations among related tasks (Baxter, 2000; Argyriou et al., 2007; Liu et al., 2017). In a sense, a very important reason why human beings can learn based on a small number of samples is that human beings can make full use of various senses to obtain enough information and synthesize relevant information. MTSL is one of the ways to realize this idea.

Classical MTSL can be roughly divided into two categories, namely, MTSL based on constraint sharing and MTSL based on parameter sharing. In relation to the first method, Argyriou et al. (2008) proposed the MTL-*L*_21_ based on regularization strategies, that was achieved by adding a regularization term for all the tasks’ objective function coefficients on the cost function. But this method performs poorly when data features have the problem of collinearity. To reduce the impact of this problem, Chen et al. (2012) added a quadratic regularization term for all the tasks’ objective function coefficients based on MTL-*L*_21_. In 2015, Duong et al. (2015) used *L*_2_ distance to regularize the parameters in their multi-task neural networks, so that each task has similar but different model parameters. In 2017, Yang and Hospedales (2017) used the trace norm to implement Duong’s model. In 2019, Oliveira et al. (2019) attempted to conceive a group LASSO with asymmetric transference formulation in multi-task learning, looking for the best of both worlds in a framework that admits the overlap of groups. Since all of these MTSL methods need to learn sparse features, their performance is not ideal when the data has few features. The MTSL methods based on parameter sharing (Caruana, 1997; Jacob et al., 2009) are not affected by this problem. In 1997, Caruana (Caruana, 1997) proposed a MTSL method (MTL) based on backpropagation neural networks. He mirrored the correlation information by sharing the input and hidden layer neurons among different tasks. In 1998, Lecun et al. (1998) used convolutional neural networks, named as LeNet-5, for document recognition on the basis of MTL. Their results clearly demonstrated the advantages of training a recognizer at the word level, rather than training it on presegmented, hand-labeled, isolated characters. In 2018, Ma et al. (2018) proposed multi-gate mixture-of-experts (MMoE), which adapted the mixture-of-experts (MoE) structure to multi-task learning by sharing the expert submodels across all tasks, while also had a gating network trained to optimize each task. In 2021, Zhang et al. (Zhang et al., 2021) developed a programming framework, AutoMTL, which generates compact multi-task models given an arbitrary input backbone convolutional neural network model and a set of tasks. However, these methods have high computational complexity and poor learning performance when the training samples are insufficient.

To address the aforementioned problems, this paper proposes a MTSL method based on a constraint sharing framework of stochastic configuration networks (SCNs) proposed by Wang et al. (Wang and Li, 2017a; Wang and Li, 2017b) Instead of the complex gradient descent method for solving the weight parameters of hidden layer nodes in general neural networks, SCNs use a supervision mechanism to stochastically configure these parameters. This stochastic configuration mechanism greatly reduces the computational complexity. Inspired by this idea, we establish a multi-task supervised learning algorithm based on stochastic configuration radial basis networks (MTSL-SCRBN). The main contributions of this study are as follows.

1. We combine constraint sharing of SCNs and parameter sharing of MTSL organically. The shared parameters are stochastically configured under certain constraint, which has low computational complexity. At the same time, to improve learning performance, the radial basis functions (Powell, 1987; Broomhead and Lowe, 1988) with different scale parameters are used as the basis functions to replace the original sigmoid functions of SCNs.
2. Two types of difficult to choice hyper parameters of the proposed model, the scale parameters and the centers of the radial basis functions, are stochastically configured during the learning process.

The rest of the paper is organized as follows. In Section 2, we briefly review MTL-*L*_21_, MTEN, MTL, and SCNs. Section 3 details our proposed algorithm MTSL-SCRBN and proves its convergence. The experimental results of these algorithms on one simulation data set and five real data sets are detailed in Section 4. Section 5 summarizes this paper.

## 2 Related Work

Firstly, we introduce some notations. Suppose that there are *M* supervised learning tasks. The samples of the *m*-th task are given by,

$$\left\{\left(x_{1}^{m},y_{1}^{m}\right),\ldots ,\left(x_{N_{m}}^{m},y_{N_{m}}^{m}\right)\right\},$$ (1)

where $x_{i}^{m}={\left[x_{i,1}^{m},\ldots ,x_{i,d}^{m}\right]}^{\text{T}}\in R^{d}$ , $y_{i}^{m}\in R$ , *i* = 1, …, *N*_*m*_, *m* = 1, …, *M*, and T means transpose transform.

### 2.1 Multi-Task Learning Methods Based on Constraint Sharing

Inspired by group sparsity, Argyrios et al. (Argyriou et al., 2008) proposed the MTL-*L*_21_ method to learn the correlation among multiple tasks under a regularization strategy. It can be described as the following optimization problem,

$$V_{MTL-L_{21}}^{*}=\underset{V\in R^{d\times M}}{\mathrm{arg min}}\overset{M}{\underset{m=1}{\sum }}\|X^{m}v^{m}-y^{m}{\|}_{F}^{2}+\lambda \|V{\|}_{2,1},$$

where $X^{m}={\left[x_{1}^{m},\ldots ,x_{N_{m}}^{m}\right]}^{\text{T}}\in R^{N_{m}\times d}$ , $y^{m}={\left[y_{1}^{m},\ldots ,y_{N_{m}}^{m}\right]}^{\text{T}}\in R^{N_{m}}$ , ‖ ⋅‖_*F*_ is the Frobenius norm, *V* = [**v** ^1^, …, **v** ^*M*^] on behalf of the model coefficient matrix, $v^{m}={\left[v_{1}^{m},\ldots ,v_{d}^{m}\right]}^{\text{T}}$ represents the *m*-th column of *V*, which is the coefficient vector of the *m*-th task. *λ* represents the regularization coefficient and $\|V{\|}_{2,1}=\sum_{i=1}^{d}\sqrt{\sum_{m=1}^{M}{\left(v_{i}^{m}\right)}^{2}}$ . For the input ${\tilde{x}}^{m}$ of the *m*-th task, MTL-*L*_21_ gives the predicted value $f\left({\tilde{x}}^{m}\right)={\tilde{x}}^{m^{\text{T}}}v_{MTL-L_{21}}^{m*}$ .

When data features have the problem of collinearity, MTL-*L*_21_ will have an unstable prediction performance. Xi Chen et al. (Chen et al., 2012) proposed the MTEN method by adding another quadratic regularization term for the objective function coefficients of all tasks on the basis of MTL-*L*_21_. It can be described as the following optimization problem,

$$V_{\mathrm{MTEN}}^{*}=\underset{V\in R^{d\times M}}{\mathrm{arg min}}\overset{M}{\underset{m=1}{\sum }}\frac{1}{2n}\|X^{m}v^{m}-y^{m}{\|}_{F}^{2}+\lambda \rho \|V{\|}_{2,1}+\frac{\lambda \left(1-\rho \right)}{2}\|V{\|}_{F}^{2},$$

where *ρ* ∈ [0, 1] represents the elastic net mixing parameter. For the input ${\tilde{x}}^{m}$ of the *m*-th task, MTEN gives the predicted value $f\left({\tilde{x}}^{m}\right)={\tilde{x}}^{m^{\text{T}}}v_{\mathrm{MTEN}}^{m*}$ .

In the case of insufficient data features and data size, the two algorithms MTL-*L*_21_ and MTEN cannot obtain enough information by learning sparse features, which leads to poor prediction performance.

### 2.2 Multi-Task Learning Methods Based on Parameter Sharing

Caruana (1997) implemented MTSL on backpropagation nets by sharing input and hidden layer neurons among different tasks. Essentially, this method optimizes the choice of function space by the correlation among tasks and obtains better internal weight parameters.

**FIGURE 1:**
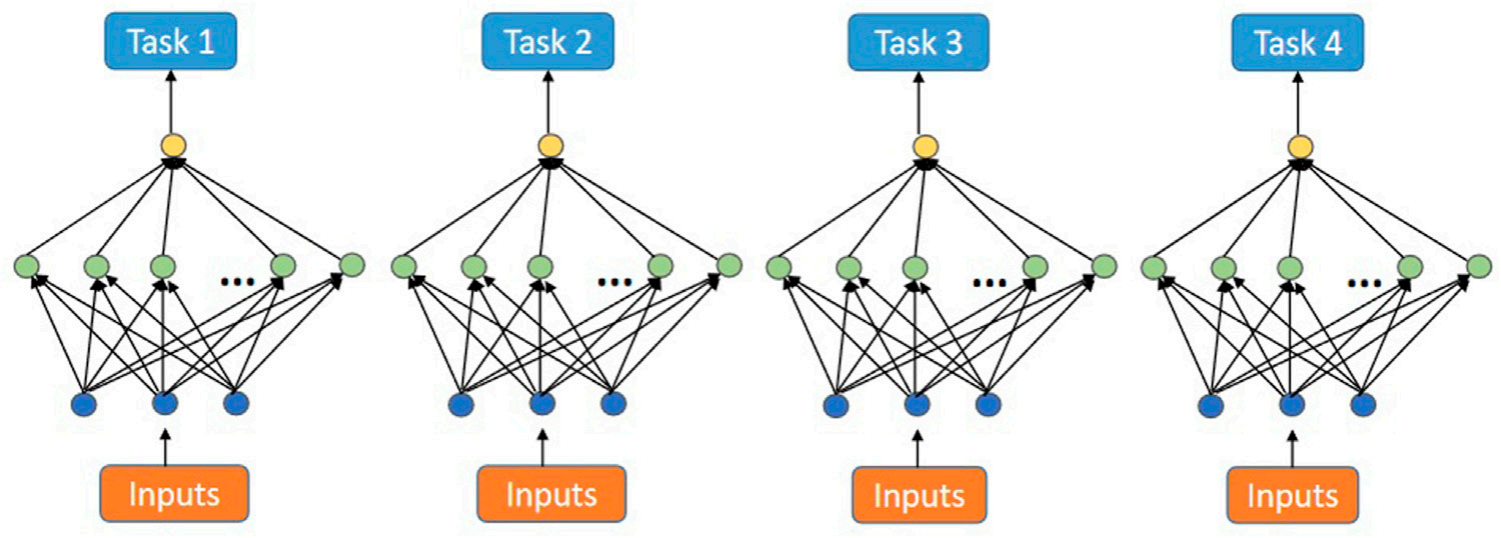
Single-task backpropagation nets.

Figure 1 shows the process of traditional backpropagation nets to deal with four related tasks. This method ignores the information among related tasks. Especially in the case of insufficient data samples, these models may have problems such as over-fitting.

**FIGURE 2:**
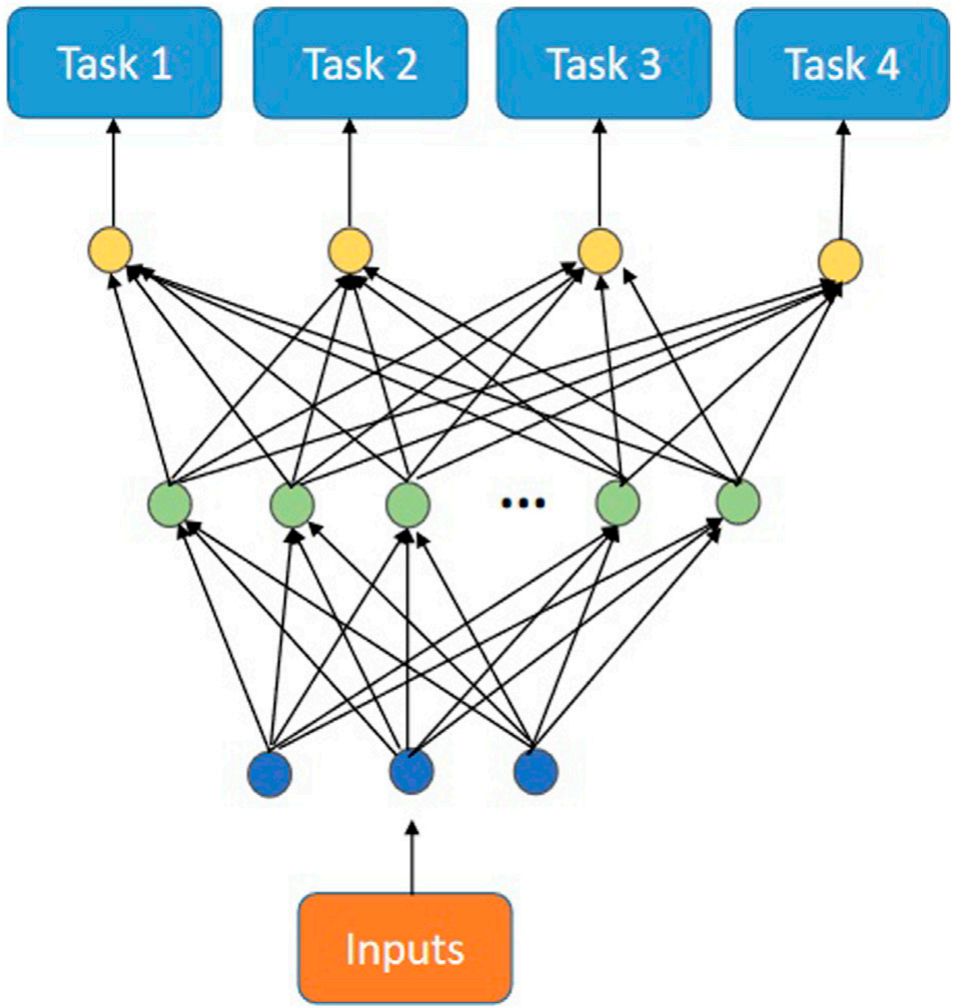
The multi-task backpropagation net.

Figure 2 shows the multi-task backpropagation net (MTL) conceived by Caruana. In MTL, each task shares input and hidden layer neurons.

Compared with the data form given in Eq. 1, the data form suitable for MTL is that different tasks have the same input,

$$X^{1}=\ldots =X^{M}=X={\left[x_{1},\ldots ,x_{N}\right]}^{\text{T}}\in R^{N\times d},$$

and the output is,

$$Y=\left[y^{1},\ldots ,y^{M}\right]\in R^{N\times M},y^{m}={\left[y_{1}^{m},\ldots ,y_{N}^{m}\right]}^{\text{T}}\in R^{N}.$$

MTL can be described as follows

$$\left(\beta^{*},W^{*},b^{*}\right)=\underset{b\in R^{S}}{\underset{\beta \in R^{S\times M}W\in R^{S\times d}}{\text{arg min}}}\overset{M}{\underset{m=1}{\sum }}\left\|y^{m}-\overset{S}{\underset{j=1}{\sum }}g_{j}\beta_{j}^{m}\right\|,$$

where *S* is the number of hidden layer nodes, $\beta_{j}^{m}$ is the external weight parameter of the *m*-th task in the *j*-th hidden layer node. $g_{j}≔g_{j}\left(X\right)={\left[g\left(x_{1}^{\text{T}}w_{j}+b_{j}\right),\ldots ,g\left(x_{N}^{\text{T}}w_{j}+b_{j}\right)\right]}^{\text{T}}$ (here *g* represents the sigmoid function), $w_{j}={\left[w_{j,1},\ldots ,w_{j,d}\right]}^{\text{T}}$ and *b*_*j*_ represent the internal weight parameter vector and the bias internal weight parameter shared by the backpropagation net. ***β*** ∈ *R* ^*N*×*M*^, *W* ∈ *R* ^*d*×*N*^, **b** ∈ *R* ^*N*^ are the corresponding parameter matrix. For the input ${\tilde{x}}^{m}$ of the *m*-th task, MTL gives the predicted value $f\left({\tilde{x}}^{m}\right)=\sum_{j=1}^{S}g\left({\tilde{x}}^{m^{\text{T}}}w_{j}^{*}+b_{j}^{*}\right)\beta_{j}^{m*}$ .

From a mathematical point of view, the essence of the backpropagation net is the gradient descent algorithm. In single-task supervised learning, the backpropagation net may fall into local optimum. However, in multi-task supervised learning, the local optimum of different tasks is in different positions, and the interaction among tasks can help the hidden layer to escape from local optimums (Caruana, 1997).

### 2.3 Stochastic Configuration Networks

Wang and Li (Wang and Li, 2017a; Wang and Li, 2017b) proposed supervised stochastic configuration networks, and implemented SCNs using three algorithms SC-i (i=I,II,III). SC-i starts with a small network structure (Tin-Yan Kwok and Dit-Yan Yeung, 1997), and uses a supervision mechanism to add hidden layer neurons until the model meets a predetermined error criterion. Since SC-III performs the best of the three algorithms, we next describe the implementation of the SC-III algorithm.

Suppose a SC-III with *L* − 1 hidden layer nodes has already been constructed, that is,

$$f^{L-1}\left(x^{\text{T}}\right)=\overset{L-1}{\underset{j=1}{\sum }}g\left(x^{\text{T}}w_{j}^{*}+b_{j}^{*}\right)\beta_{j}^{L-1},\left(L=2,3,\ldots ,f^{0}\in \left[0,0,\ldots ,0\right]\in R^{q}\right),$$

where $\beta_{j}^{L-1}=\left[\beta_{j,1}^{L-1},\ldots ,\beta_{j,q}^{L-1}\right]\in R^{q}$ represents the optimal external weight parameter of the *j*-th hidden layer node, $w_{j}^{*}$ and $b_{j}^{*}$ represent the optimal internal weight parameters of the *j*-th hidden layer node.

For training data set $X={\left[x_{1},\ldots ,x_{N}\right]}^{\text{T}}\in R^{N\times d}$ , *Y* = [**y** ^1^, …, **y** ^*q*^] ∈ *R* ^*N*×*q*^, where $x_{i}={\left[x_{i,1},\ldots ,x_{i,d}\right]}^{\text{T}}$ . Let $f^{L-1}≔f^{L-1}\left(X\right)=\sum_{j=1}^{L-1}g_{j}\left(X\right)\beta_{j}^{L-1}$ and $e^{L-1}≔Y-f^{L-1}≔\left[e_{1}^{L-1},\ldots ,e_{q}^{L-1}\right]$ , which is the residual error matrix of the (*L* − 1)-th hidden layer node. If ‖ ***e*** ^*L*−1^‖_*F*_ does not meet the predetermined error criteria, SC-III needs to generate a new hidden layer node, that is, stochastically configure internal weight parameters **w**_*L*_, *b*_*L*_ from an uniform distribution *U* ^*d*+1^[ − Φ, Φ], Φ > 0. For new variables,

$$\xi_{n}^{L}=\frac{{\left\langle e_{n}^{L-1},g_{L}\right\rangle }^{2}}{\|g_{L}{\|}_{2}^{2}}-\left(1-r-\mu_{L}\right)\|e_{n}^{L-1}{\|}_{2}^{2},\;n=1,\ldots ,q,$$

if $\underset{n}{\min}\left(\xi_{n}^{L}\right)\geq 0$ , then **w**_*L*_, *b*_*L*_ are considered to meet the condition, otherwise **w**_*L*_, *b*_*L*_ need to be configured again. With the qualified internal weight parameters $w_{L}^{*}$ and $b_{L}^{*}$ , SC-III obtains the optimal external weight parameter vector by the following optimization problem,

$$\beta^{L}=\underset{\beta \in R^{L\times q}}{\mathrm{arg min}}{\left\|Y-\overset{L}{\underset{j=1}{\sum }}g_{j}\beta_{j}\right\|}_{F}^{2}.$$

The leading model $f^{L}\left(x\right)=\sum_{j=1}^{L}g\left(x^{\text{T}}w_{j}^{*}+b_{j}^{*}\right)\beta_{j}^{L}$ will have an improved residual error. Repeat the above steps to add hidden layer nodes until the residual error meets the predetermined error criteria.

## 3 Multi-Task Supervised Learning Based on Stochastic Configuration Radial Basis Networks

In this section, we introduce the proposed MTSL-SCRBN algorithm.

### 3.1 Model Introduction

In order to combine SCNs and MTL organically, we need to change the data form given in Eq. 1. In MTSL-SCRBN, first, we require each task to have a same number of samples, namely, *N*_1_ =…= *N*_*M*_ =: *N*. (If the number of samples for each task is different, this requirement can be achieved by random sampling.) Then we merge the input data of different tasks into a new input data, that is, the *i*-th new input data is $X_{i}^{\text{T}}:=\left(x_{i}^{1^{\text{T}}},\ldots ,x_{i}^{M^{\text{T}}}\right)\in R^{1\times Md}$ , where $x_{i}^{m}\in R^{d}$ is the *i*-th input of the *m*-th task, for *i* = 1, …, *N* and *m* = 1, …, *M*. The corresponding *i*-th new output data is $y_{i}:={\left(y_{i}^{1},\ldots ,y_{i}^{M}\right)}^{T}$ , where $y_{i}^{m}$ is the original output of $x_{i}^{m}$ in the *m*-th task. The goal of our MTSL-SCRBN is to establish an appropriate model from *R* ^*Md*^ to *R* ^*M*^ based on these data $\left\{{\left(X_{i},y_{i}\right)}_{i=1}^{N}\right\}$ .

In order to obtain good learning performance, we use the following radial basis function *k*_*σ*_(**x**, **x**′) as model’s basis function,

$$k_{\sigma }\left(x,x^{\prime }\right)=\exp\left(-\frac{\|x-x^{\prime }{\|}^{2}}{2\sigma^{2}}\right),$$

where **x** is the input, **x**′ represents the center and *σ* is the scale parameter.

Suppose *f* ^0^ = [0, 0, …, 0] ∈ *R* ^*M*^, for *L* = 2, 3, …, it is assumed that a MTSL-SCRBN with *L* − 1 hidden layer nodes has already been constructed as follows,

$$f^{L-1}\left(X\right)=\overset{L-1}{\underset{j=1}{\sum }}\left[k_{\sigma_{j}^{*}}\left(x^{1},x^{j*}\right),\ldots ,k_{\sigma_{j}^{*}}\left(x^{M},x^{j*}\right)\right]\beta_{j}^{L-1},$$

where $X^{\text{T}}=\left(x^{1^{\text{T}}},\ldots ,x^{M^{\text{T}}}\right)$ is a new input formed by the inputs of *M* tasks, and $\beta_{j}^{L-1}=\left[\beta_{j}^{1,L-1},\ldots ,\beta_{j}^{M,L-1}\right]\in R^{M\times M}$ ,where $\beta_{j}^{m,L-1}\in R^{M}$ represents the optimal external weight parameter vector of the *m*-th task in the *j*-th hidden layer node, **x** ^j^* and $\sigma_{j}^{*}$ are the optimal center and the optimal scale parameter of the radial basis function in the *j*-th hidden layer node, respectively. Different from the traditional learning of radial basis neural network, in our MTSL-SCRBN, the optimal centers and the optimal scale parameters at each step are randomly assigned by a shared supervision mechanism given in the following. This is simple to implement and easy to obtain a learning model with good performance.

Denote $k_{j}^{m}≔{\left[k_{\sigma_{j}^{*}}\left(x_{1}^{m},x^{j*}\right),\ldots ,k_{\sigma_{j}^{*}}\left(x_{N}^{m},x^{j*}\right)\right]}^{\text{T}},K_{j}≔\left[k_{j}^{1},\ldots ,k_{j}^{M}\right]\in R^{N\times M}$ and $f^{L-1}≔\sum_{j=1}^{L-1}K_{j}\beta_{j}^{L-1}$ , $Y={\left[y_{1},\ldots ,y_{N}\right]}^{\text{T}}\in R^{N\times M}$ . Then, let ***e*** ^*L*−1^≔*Y* − *f* ^*L*−1^≔[**e** ^1,*L*−1^, …, **e** ^*M*,*L*−1^] be the residual error matrix of the (*L* − 1)-th hidden layer node. If ‖ ***e*** ^*L*−1^‖_*F*_ does not meet the predetermined error criteria, MTSL-SCRBN needs to generate a new hidden layer node, that is, stochastically configure the scale parameter *σ*_*L*_ from *U*[0, Ω], Ω > 0 and the center of the radial basis function **x** ^*L*^ from $\left\{x_{i}^{m}:i=1,\ldots ,N,\;m=1,\ldots ,M\right\}$ .

Similar to that in SCNs, we introduce a variable *ξ* ^*m*,*L*^ in our multi-task learning case as follows,

$$\xi^{m,L}=\frac{1}{M}\frac{{\left\langle e^{m,L-1},k_{L}^{m}\right\rangle }^{2}}{\|k_{L}^{m}{\|}_{2}^{2}}-\left(1-r-\mu_{L,r}\right)\|e^{m,L-1}{\|}_{2}^{2}.$$

Here $k_{L}^{m}={\left[k_{\sigma_{L}}\left(x_{1}^{m},x^{L}\right),\ldots ,k_{\sigma_{L}}\left(x_{N}^{m},x^{L}\right)\right]}^{\text{T}}$ , 0 < *r* < 1 is a given constant and $\mu_{L,r}=\frac{1-r}{L+1}$ .

If $\sum_{m=1}^{M}\xi^{m,L}\geq 0$ , then *σ*_*L*_, **x** ^*L*^ are considered to meet the condition, otherwise, *σ*_*L*_, **x** ^*L*^ need to be configured again. With the qualified parameters $\sigma_{L}^{*}$ and **x** ^*L*∗^, MTSL-SCRBN obtains the optimal external weight parameter vector by the following optimization problem,

$$\beta^{L}=\underset{\beta \in R^{LM\times M}}{\mathrm{arg min}}{\left\|Y-\overset{L}{\underset{j=1}{\sum }}K_{j}\beta_{j}\right\|}_{F}^{2}.$$

The leading model,

$$f^{L}\left(X\right)=\overset{L}{\underset{j=1}{\sum }}\left[k_{\sigma_{j}^{*}}\left(x^{1},x^{j*}\right),\ldots ,k_{\sigma_{j}^{*}}\left.\left(x^{M},x^{j*}\right)\right)\right]\beta_{j}^{L},$$

will have an improved residual error. Repeat the above steps to add hidden layer nodes until the residual error meets the predetermined error criteria.

The above implementation process of the proposed MTSL-SCRBN algorithm is described as follows.

Algorithm 1 The MTSL-SCRBN algorithm

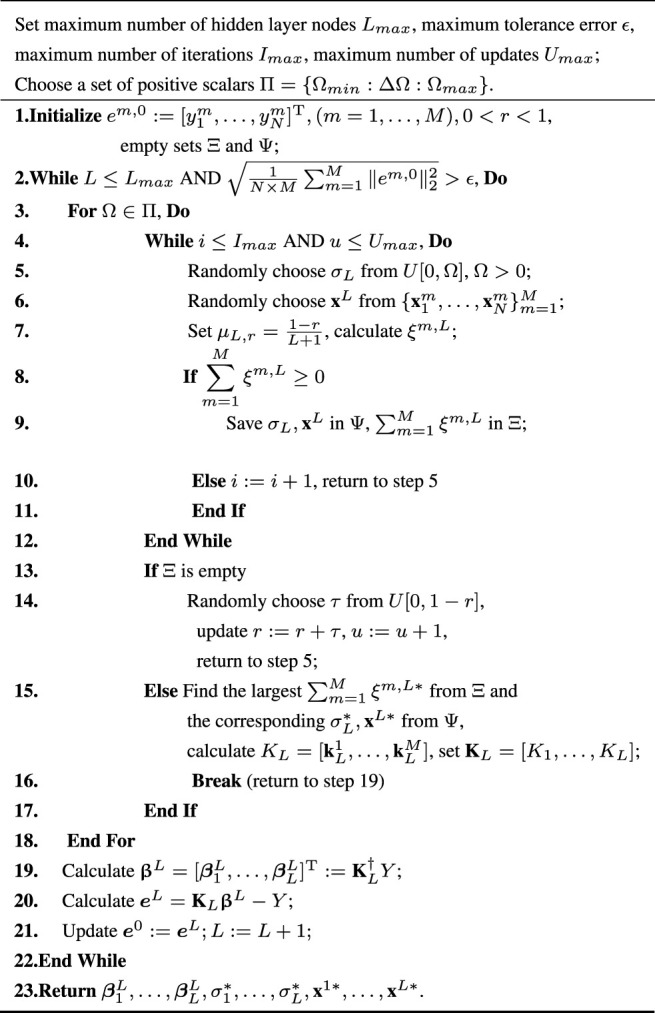

Notice that in step 19, we calculate the parameter matrix based on the standard least squares method,

$$\beta^{L}=\underset{\beta \in R^{LM\times M}}{\mathrm{arg min}}{\left\|Y-\overset{L}{\underset{j=1}{\sum }}K_{j}\beta_{j}\right\|}_{F}^{2}=K_{L}^{†}Y,$$

where $K_{L}^{†}$ is the Moore-Penrose generalized inverse (Lancaster and Tismenetsky, 1985) of **K**_*L*_. The setting of *μ*_*L*,*r*_ in step 7 and the updating idea of *r* in step 14 can be referred to literature (Wang and Li, 2017a).

### 3.2 The Convergence Theorem of the MTSL-SCRBN Algorithm

We extend the method in (Wang and Li, 2017a) to the multi-task learning framework of this paper and prove the convergence of the proposed algorithm.

Theorem 1. Assume that there are some *p*_**k**_ ∈ *R* ^+^, satisfying $0<\|k_{j}^{m}{\|}_{2}<p_{k}$ . Given 0 < *r* < 1 and a non-negative real value sequence {*μ* *_L_*} with lim_*L*→+∞_ *μ* *_L_* = 0 and *μ*_*L*_ ≤ (1 − *r*). For *L* = 2, 3 …, denoted by

$$\delta^{L}=\overset{M}{\underset{m=1}{\sum }}\delta^{m,L},$$

$$\delta^{m,L}=\left(1-r-\mu_{L}\right)\|e^{m,L-1}{\|}_{2}^{2}.$$

If the basis function $k_{L}^{m}$ is generated to satisfy the following inequality,

$$\overset{M}{\underset{m=1}{\sum }}{\left\langle e^{m,L-1},k_{L}^{m}\right\rangle }^{2}\geq \frac{M^{2}}{2M-2}p_{k}^{2}\delta^{L},$$ (2)

and the external weight parameter vector is evaluated by,

$$\beta^{L}={\left[\beta_{1}^{L},\ldots ,\beta_{L}^{L}\right]}^{\text{T}}=\underset{\beta \in R^{LM\times M}}{\mathrm{arg min}}{\left\|Y-\overset{L}{\underset{j=1}{\sum }}K_{j}\beta_{j}\right\|}_{F}^{2}.$$

*Then, we have* lim_*L*→+*∞*_‖*Y* − *f* ^*L*^‖_*F*_ = 0*.*

Proof of Theorem 1. Define intermediate val*ues*

$${\tilde{\beta }}_{L}^{m,L}={\left[\frac{\langle e^{m,L-1},k_{L}^{1}\rangle }{M\|k_{L}^{1}{\|}_{2}^{2}},\ldots ,\frac{\langle e^{m,L-1},k_{L}^{M}\rangle }{M\|k_{L}^{M}{\|}_{2}^{2}}\right]}^{\text{T}},$$

*and* ${\tilde{e}}^{L}=e^{L-1}-K_{L}{\tilde{\beta }}_{L}^{L}$ *, with* ${\tilde{\beta }}_{L}^{L}=\left[{\tilde{\beta }}_{L}^{1,L},\ldots ,{\tilde{\beta }}_{L}^{M,L}\right],e^{0}=Y$ *.*

*It is clear that* $\|e^{L}{\|}_{F}^{2}\;\leq \;\|{\tilde{e}}^{L}{\|}_{F}^{2}\;\leq \;\|e^{L-1}{\|}_{F}^{2}\;\leq \;\|{\tilde{e}}^{L-1}{\|}_{F}^{2}$ *, where* *L* = 2, 3, …*. So* $\left\{\|e^{L-1}{\|}_{F}^{2}\right\}$ *is monotonically decreasing and convergent. Hence, we have*,

$$\begin{array}{ll} & \|e^{L}{\|}_{F}^{2}-\left(r+\mu_{L}\right)\|e^{L-1}{\|}_{F}^{2}\\ \leq & \|{\tilde{e}}^{L}{\|}_{F}^{2}-\left(r+\mu_{L}\right)\|e^{L-1}{\|}_{F}^{2}\\ = & \overset{M}{\underset{m=1}{\sum }}\left(\left\langle e^{m,L-1}-K_{L}{\tilde{\beta }}_{L}^{m,L},e^{m,L-1}-K_{L}{\tilde{\beta }}_{L}^{m,L}\right\rangle -\left(r+\mu_{L}\right)\left\langle e^{m,L-1},e^{m,L-1}\right\rangle \right)\\ = & \left(1-r-\mu_{L}\right)\|e^{L-1}{\|}_{F}^{2}-\overset{M}{\underset{m=1}{\sum }}\left(2\left\langle e^{m,L-1},K_{L}{\tilde{\beta }}_{L}^{m,L}\right\rangle -\left\langle K_{L}{\tilde{\beta }}_{L}^{m,L},K_{L}{\tilde{\beta }}_{L}^{m,L}\right\rangle \right)\\ \leq & \left(1-r-\mu_{L}\right)\|e^{L-1}{\|}_{F}^{2}\;-\;\frac{2M-2}{M^{2}}\;\overset{M}{\underset{m=1}{\sum }}\left(\;\frac{{\left\langle e^{m,L-1},k_{L}^{1}\right\rangle }^{2}}{\|k_{L}^{1}{\|}_{2}^{2}}+\cdots +\frac{{\left\langle e^{m,L-1},k_{L}^{M}\right\rangle }^{2}}{\|k_{L}^{M}{\|}_{2}^{2}}\;\right)\\ \leq & \left(1-r-\mu_{L}\right)\|e^{L-1}{\|}_{F}^{2}-\frac{2M-2}{M^{2}}\overset{M}{\underset{m=1}{\sum }}\frac{{\left\langle e^{m,L-1},k_{L}^{m}\right\rangle }^{2}}{\|k_{L}^{m}{\|}_{2}^{2}}\\ = & \delta^{L}-\frac{2M-2}{M^{2}}\overset{M}{\underset{m=1}{\sum }}\frac{{\left\langle e^{m,L-1},k_{L}^{m}\right\rangle }^{2}}{\|k_{L}^{m}{\|}_{2}^{2}}\\ \leq & \delta^{L}-\frac{2M-2}{M^{2}}\frac{\sum_{m=1}^{M}{\left\langle e^{m,L-1},k_{L}^{m}\right\rangle }^{2}}{p_{k}^{2}}\\ \leq & 0. \end{array}$$

Then, the following inequality holds,

$$\|e^{L}{\|}_{F}^{2}\leq r\|e^{L-1}{\|}_{F}^{2}+\gamma_{L},\left(\gamma_{L}=\mu_{L}\|e^{L-1}{\|}_{F}^{2}\geq 0\right).$$

*Since* lim_*L*→+*∞*_ *μ*_*L*_ = 0*, and* 0 < *r* < 1*, we have* $\lim_{L\to +\infty }\|e^{L}{\|}_{F}^{2}=0$ *, and* lim_*L*→+*∞*_‖ ***e*** ^*L*^‖_*F*_ = 0*.*

Remark 1. Unli*ke SC-III, we relax the condition for the configuration parameters in the formula* (∗)*. SC-III requires each task to meet the inequality conditions, but MTSL-SCRBN only requires the sum of all tasks to satisfy the inequality condition. The rationality of this condition will also be verified in the experiment results of next section.*

## 4 Experiment Results

In order to show the effectiveness of the proposed algorithm, this section uses the classical STSL algorithms SVM (Cortes and Vapnik, 1995), SC-III (Wang and Li, 2017a) and seven MTSL algorithms MTSL-SCRBN, MTL (Caruana, 1997), MTEN (Chen et al., 2012), DMTRL (Yang and Hospedales, 2017), MMoE (Ma et al., 2018), GAMTL (Oliveira et al., 2019), AUTOMTL (Zhang et al., 2021) to perform comparative experiments. All calculations are conducted using Python 3.6.5 on a computer with 2.60 GHz CPU and 8 GB RAM. The input features are scaled into [ − 1, 1] and the output remains unchanged. All the results reported in this paper take averages over 20 independent trials, except for the SVM and MTEN algorithms, which have fixed experiment results. The accuracy (ACC) and root mean square error (RMSE) are chosen as the classification and regression evaluation indicators, where

$$RMSE={\left[\frac{1}{N\times M}\overset{M}{\underset{m=1}{\sum }}\overset{N}{\underset{i=1}{\sum }}{\left({\hat{y}}_{i}^{m}-y_{i}^{m}\right)}^{2}\right]}^{\frac{1}{2}},$$

with $y_{i}^{m}$ and ${\hat{y}}_{i}^{m}$ representing the target output and the learner’s output of *i* − th sample for task *m* respectively.

For different data sets, some algorithms used in the following experiments can stochastically configure hyperparameters within specified ranges or determine parameters by cross-validation. Table 1 gives the specific selection range of each parameter.

**TABLE 1:** Parameter description.

Algorithms	Parameters	Parameters’ Range
MTSL-SCRBN	RBF scale *σ*	*σ* ∼ *U*[0, Ω], Ω ∈ (0, 100]
MTEN	Regularization parameters *λ*, the elastic net mixing parameter *ρ*	*λ* ∈ {10^*t*^, *t* = −6, − 5.5, …, 6}, *ρ* ∈ [0, 1]
SVM	RBF scale *σ*, Penalty parameter C	*σ* ∈ {2^–5^, 2^–4^, …, 2^4^, 2^5^}, *C* ∈ {10^*t*^, *t* = −4, − 3, …, 3, 4}
SC-III	Internal weight parameters **w**, *b*	(**w**, *b*) ∼ *U* ^*d*+1^[ − Φ, Φ], Φ ∈ {1, 5, 15, 30, 50, 100, 150, 200}
DMTRL	Factorisation method parameters	{*LAF*, *Tucker*, *TT*}
MMoE	Units *u*, NumExperts *num*	*u* ∈ {5, 6, 7, …, 19, 20}, *num* ∈ {5, 6, 7, …, 19, 20}
AUTOMTL	Weight *LR*, Policy *p*, Decay *d*, Iteration *iter*	*LR* = 0.001, *p* = 0.01, *d* = 0.5, *iter* = 400

### 4.1 Experimental Results and Analysis on Simulated Data

The simulation data set selected in this paper is generated by the following five functions, which we refer as five tasks,

$$\begin{array}{ll} \text{ Task }1\text{: }f_{1}\left(x\right) & =2e^{-{\left(10x-4\right)}^{2}}+5e^{-{\left(80x-40\right)}^{2}}+3e^{-{\left(80x-20\right)}^{2}}+4e^{-{\left(90x-60\right)}^{2}}\\ \text{ Task }2\text{: }f_{2}\left(x\right) & =10e^{-{\left(10x-4\right)}^{2}}+25e^{-{\left(80x-40\right)}^{2}}+15e^{-{\left(80x-20\right)}^{2}}+20e^{-{\left(90x-60\right)}^{2}}\\ \text{ Task }3\text{: }f_{3}\left(x\right) & =18e^{-{\left(10x-4\right)}^{2}}+45e^{-{\left(80x-40\right)}^{2}}+27e^{-{\left(80x-20\right)}^{2}}+36e^{-{\left(90x-60\right)}^{2}}.\\ \text{ Task }4\text{: }f_{4}\left(x\right) & =26e^{-{\left(10x-4\right)}^{2}}+65e^{-{\left(80x-40\right)}^{2}}+39e^{-{\left(80x-20\right)}^{2}}+52e^{-{\left(90x-60\right)}^{2}}\\ \text{ Task }5\text{: }f_{5}\left(x\right) & =34e^{-{\left(10x-4\right)}^{2}}+85e^{-{\left(80x-40\right)}^{2}}+51e^{-{\left(80x-20\right)}^{2}}+68e^{-{\left(90x-60\right)}^{2}} \end{array}$$

**FIGURE 3:**
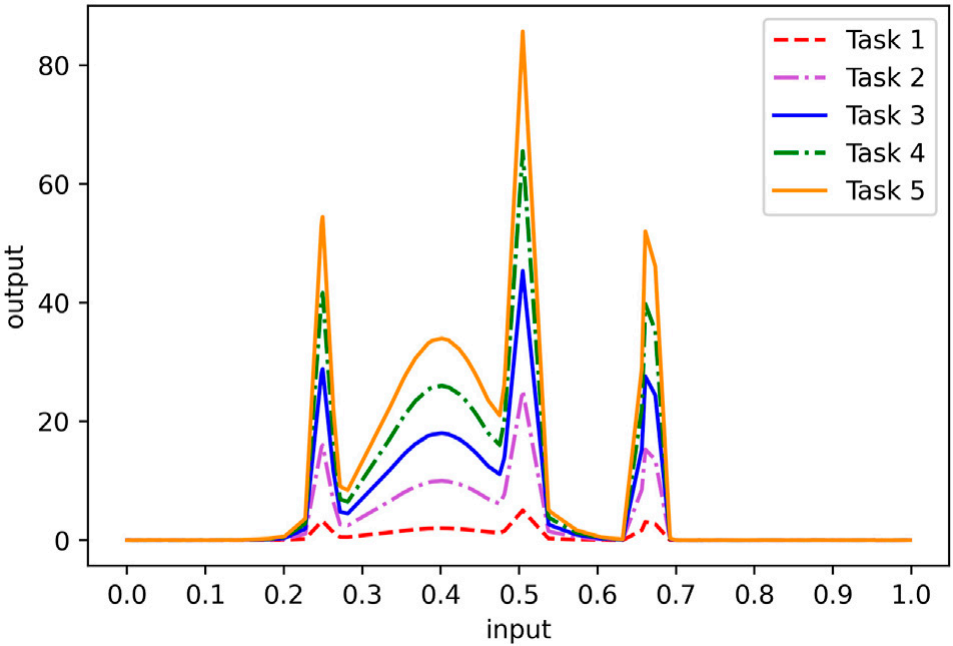
Distribution of the simulation data set.

Figure 3 depicts the distributions of five functions on [0, 1]. As we can see, when the independent variables of the five functions are the same, the function values follow similar trends. Therefore, learning the function values of five functions with the same independent variable can be regarded as a multi-task learning. Here, we independently extract 100 one-dimensional input data from the same uniform distribution, then calculate the corresponding function values according to these five functions, and add white Gaussian noise with a standard deviation of 0.01 to form 100 five-dimensional output data. In the following experiments, we randomly select 70% of the data as training data and 30% of the data as test data. From the figures of the five functions, it can be seen that only 70 training samples are not enough to achieve good single-task learning results. We verify this point by experimenting with single-task and multi-task algorithms.

**FIGURE 4:**
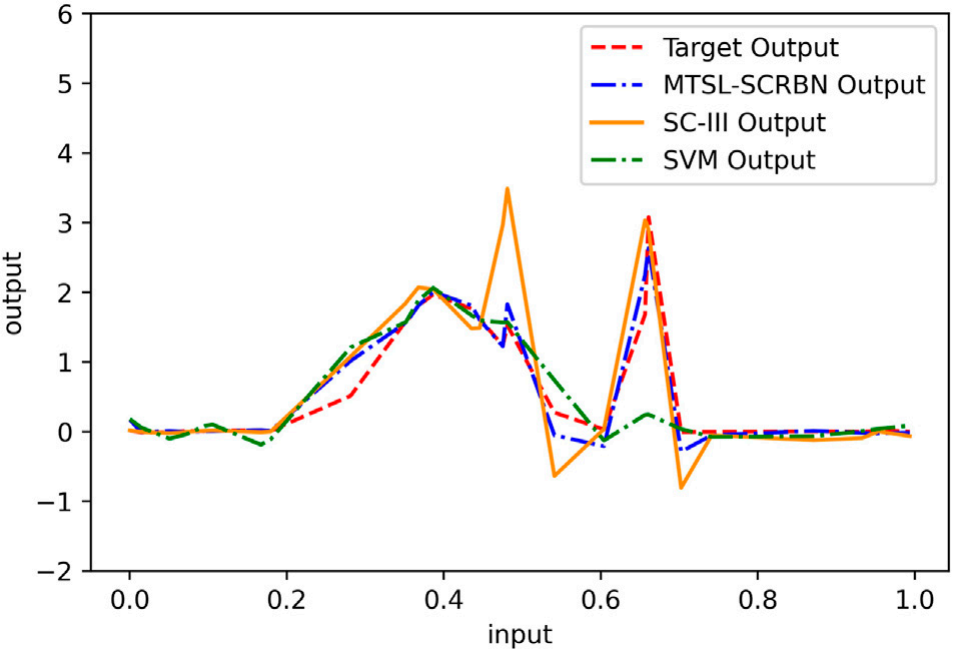
Prediction performance of MTSL-SCRBN, SC-III and SVM on Task 1 of simulation data set.

Firstly, we compare the learning performance of the proposed MTSL-SCRBN with other two STSL algorithms, SVM and SC-III, on five tasks. For MTSL-SCRBN, these five tasks are combined to learn together. The training and test RMSEs on five tasks for these three methods are given in Table 2. Clearly, the proposed multi-task learning model can product better performance on each task than the two STSL models, which only use 70 samples to learn each task independently. Furthermore, we show the learning effects of the three algorithms on Task 1 in Figure 4. It is can be seen that the proposed MTSL-SCRBN has good learning performance where the data changes dramatically.

**TABLE 2:** The results of MTSL-SCRBN, SC-III and SVM on the simulation data set.

Task	MTSL-SCRBN		SC-III		SVM	
	training	test	training	test	training	test
Task 1	0.1080	**0.2589**	0.1735	0.4264	0.6624	0.8129
Task 2	0.5320	**1.2773**	0.8595	2.1120	3.4796	4.2842
Task 3	0.9589	**2.2985**	1.5501	3.7974	6.0838	7.6899
Task 4	1.3853	**3.3163**	2.2385	5.4810	8.8058	11.1654
Task 5	1.8116	**4.3383**	2.9267	7.1716	11.5199	14.6416

The results with the minimum test errors are marked in bold.

**FIGURE 5:**
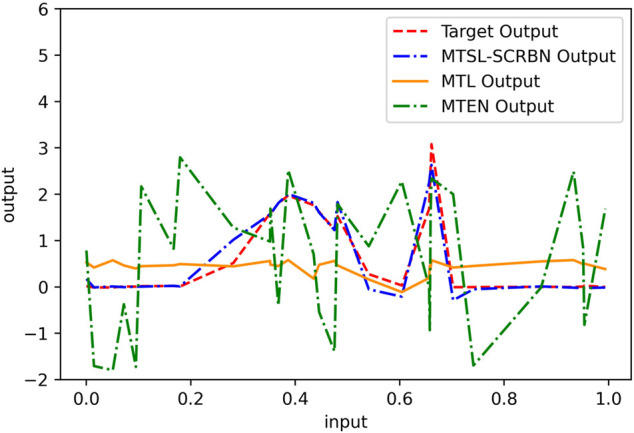
Prediction performance of MTSL-SCRBN, MTL, MTEN on Task 1 of simulation data set.

Next, the comparison results of three MTSL algorithms, MTSL-SCRBN, MTL, MTEN, on the simulation data set are recorded in Table 3 and Figure 5. In Table 3, the values in parentheses represent the standard deviations of 20 experiments’ results. According to these results, compared with MTEN and MTL, the proposed MTSL-SCRBN has better approximation ability.

**TABLE 3:** The results of MTSL-SCRBN, MTL, MTEN on the simulation data set.

MTSL-SCRBN		MTL		MTEN	
training	test	training	test	training	test
1.2836	**2.6383**	6.7430	5.1472	7.8274	6.2219
(0.1763)	(0.0929)	(0.0336)	(0.0187)		

The results with the minimum test errors are marked in bold.

### 4.2 Experimental Results and Analysis on Benchmark Datasets

This subsection further compares seven MTSL algorithms on four benchmark datasets. They are MTL, MTEN, DMTRL, MMoE, GAMTL, AUTOMTL and the proposed MTSL-SCRBN. According to the characteristics of data sets and algorithms, different algorithms will be selected for comparative analysis on different data sets. The four benchmark datasets include three regression problems on the stock portfolio performance data set, the bionic robot data set SARCOS and the School data set, one classification problem on the Mnist data set from Yann LeCun^1^ The basic information of the four datasets are summarized in Table 4.

**TABLE 4:** Descriptions of benchmark datasets.

Data Set	Size	Feature Number	Task Number
Stock	63	6	6
SARCOS	48933	21	7
School	15362	8	139
Mnist	70000	28*28	10

Firstly, we compare the performance of MTL, MTEN, DMTRL, MMoE, GAMTL and MTSL-SCRBN on different sizes of three regression data sets. For the three data sets, we randomly choose 15/30/3,500 samples outside the training set as test set, respectively. At the same time, we select 5 tasks, all of which have more than 230 samples, from 139 tasks in School data set. Table 5 below shows the specific experiment results. As we can see, the performance of each algorithm tends to be better and more stable with an increasing number of training samples. Furthermore, the proposed MTSL-SCRBN algorithm exhibits good performance even with a small number of training observations.

**TABLE 5:** The comparison results of six MSTL algorithms on three data sets.

Data set	Size	MTSL-SCRBN		MTL		MTEN		DMTRL		MMoE		GAMTL	
		training	test	training	test	training	test	training	test	training	test	training	test
Stock		0.0796	**0.1105**	0.1524	0.3016			0.1155	0.1397	0.1441	0.1649	0.0885	0.1536
	10	(0.0039)	(0.0040)	(0.0619)	(0.0667)	0.1370	0.1387	(0.0268)	(0.0089)	(0.0045)	(0.0040)	(0.0072)	(0.0018)
		0.0661	**0.0904**	0.1149	0.2207		0.0973	0.1008	0.1362	0.1577	0.0811	0.1342	
	20	(0.0050)	(0.0069)	(0.0158)	(0.0754)	0.1313	0.1379	(0.0188)	(0.0112)	(0.0091)	(0.0057)	(0.0074)	(0.0065)
		0.0542	**0.0793**	0.1055	0.1712		0.0839	0.0975	0.1282	0.1505	0.0769	0.1186	
	30	(0.0054)	(0.0078)	(0.0117)	(0.0366)	0.1380	0.1339	(0.0156)	(0.0078)	(0.0019)	(0.0010)	(0.0033)	(0.0022)
Sarcos		2.3921	**3.6667**	5.1595	5.9636		4.8021	5.4138	3.5679	4.3704	3.6894	4.5503	
	700	(0.0221)	(0.0364)	(0.0395)	(0.0895)	4.2165	4.2680	(0.1312)	(0.1128)	(0.0165)	(0.0112)	(0.0315)	(0.0489)
		2.2732	**3.1740**	4.7317	5.6063		4.2588	4.7137	2.7236	3.4125	2.9128	3.6810	
	1,400	(0.0250)	(0.0179)	(0.0342)	(0.0808)	4.1334	4.2281	(0.0610)	(0.1084)	(0.0100)	(0.0101)	(0.0147)	(0.0286)
		2.1128	**2.9291**	4.5611	5.0693		3.4181	4.0180	2.6235	2.9898	2.7503	3.0137	
	2,100	(0.0255)	(0.0392)	(0.0370)	(0.0899)	4.1552	4.1818	(0.0683)	(0.0639)	(0.0115)	(0.0123)	(0.0239)	(0.0317)
School		8.9122	**12.1368**	11.9488	13.6566		12.0246	13.2983	11.9107	13.5805	11.7439	12.3742	
	100	(0.1221)	(0.0634)	(0.1356)	(0.1284)	12.1952	13.9454	(0.1411)	(0.1155)	(0.1286)	(0.2422)	(0.1280)	(0.1560)
		8.4406	**11.5972**	11.4453	13.3710		11.7838	13.1285	11.6480	13.2517	11.3313	11.7067	
	150	(0.0736)	(0.0828)	(0.1141)	(0.1089)	11.7394	13.5306	(0.1562)	(0.1371)	(0.0907)	(0.1108)	(0.0899)	(0.0249)
		7.6262	**10.6699**	11.1923	12.9307		11.4090	12.9873	11.3628	12.9779	10.8133	11.3346	
	200	(0.1177)	(0.1067)	(0.0874)	(0.1145)	11.3253	12.8915	(0.1589)	(0.2015)	(0.0748)	(0.0612)	(0.0546)	(0.0307)

The results with the minimum test errors are marked in bold.

Then, in order to further verify the performance of MTSL-SCRBN for classification cases, we compare the results of MTSL-SCRBN, DMTRL, MMoE, GAMTL and AUTOMTL on the Mnist data set. We randomly choose 50/100/150 samples from each task in the Mnist data set as training set, and 10000 samples in the remaining samples as test set. Considering that this is a high dimensional small sample problem, we firstly reduce the dimensionality of the data set, and then use MTSL-SCRBN for training and prediction. There are many dimensionality reduction methods, such as Principal Component Analysis(PCA) (Pearson, 1901), Latent Dirichlet Allocation(LDA) (Blei et al., 2003), Sequential Markov Blanket Criterion (SMBC) (Pratama et al., 2017), Auto Encoder (Hinton and Salakhutdinov, 2006) and so on. Here, we use the performance of the dimensionality-reduced data in the MTSL-SCRBN as the selection criterion, and choose Auto Encoder to reduce the dimension of original data set into 30 dimensions. It can be seen from Table 6 that the performance of MTSL-SCRBN which uses dimensionality reduction data set is a little bit better than that of other Multi-task deep learning algorithms, but as the sample size increases, the performance of the two algorithms gradually approaches.

**TABLE 6:** The accuracy of MTSL-SCRBN, DMTRL, MMoE, GAMTL and AUTOMTL on Mnist data set.

Size	MTSL-SCRBN		DMTRL		MMoE		GAMTL		AUTOMTL	
	training	test	training	test	training	test	training	test	training	test
	92.00%	**67.14** **%**	91.80.00%	64.67%	94.16%	62.22%	69.47%	57.01%	93.26%	60.78%
50	(0.0065)	(0.0067)	(0.0111)	(0.0099)	(0.0077)	(0.0086)	(0.0303)	(0.0124)	(0.0231)	(0.0212)
	96.15%	**72.25** **%**	95.90%	70.793%	95.92%	68.15%	84.62%	68.51%	94.56%	70.45%
100	(0.0067)	(0.0066)	(0.0117)	(0.0101)	(0.0084)	(0.0061)	(0.0144)	(0.0158)	(0.0128)	(0.0094)
	97.27%	**82.49** **%**	97.97%	80.05%	97.04%	74.61%	95.52%	78.89%	96.87%	79.34%
150	(0.0099)	(0.0064)	(0.0082)	(0.0097)	(0.0081)	(0.0063)	(0.0119)	(0.0087)	(0.0097)	(0.0084)

The results with the minimum test errors are marked in bold.

### 4.3 Comparison Experiment Results for Different Activation Functions

The previous results show that the proposed MTSL-SCRBN algorithm is effective for multi-task learning in the case of small samples. This subsection mainly discusses the impact of selecting different activation functions on algorithm performance. Here we select other three usually used activation functions. They are sigmoid function, Tanh function and ReLU function. After replacing the radial basis functions in MTSL-SCRBN with these three functions respectively, the model names are respectively called MTSL-SCSGM, MTSL-SCTANH and MTSL-SCReLU. We choose to conduct comparative experiments on the Stock and SARCOS data sets.

For different data sets, the parameters contained in each algorithm need to be randomly set or cross verified within a certain range. The specific selection range of each parameter is given in Table 7.

**TABLE 7:** Parameter description for the four models.

Models	Parameters	Parameters’ Range
MTSL-SCRBN	RBF scale *σ*	*σ* ∼ *U*[0, Ω], Ω ∈ (0, 100]
MTSL-SCSGM	Internal weight parameters **w**, *b*,	(**w**, *b*) ∈ ∼ *U* ^*d*+1^[ − Φ, Φ], Φ ∈ {1, 5, 15, 30, 50, 100, 150, 200}
MTSL-SCTANH	Internal weight parameters **w**, *b*,	(**w**, *b*) ∈ ∼ *U* ^*d*+1^[ − Φ, Φ], Φ ∈ {1, 5, 15, 30, 50, 100, 150, 200}
MTSL-SCReLU	Internal weight parameters **w**, *b*	(**w**, *b*) ∼ *U* ^*d*+1^[ − Φ, Φ], Φ ∈ {1, 5, 15, 30, 50, 100, 150, 200}

Table 8 depicts the RMSE results of stochastic configuration multi-task learning models based on four different activation functions. Under the training samples with different sample sizes in the two data sets, the MTSL-SCRBN, which based on radial basis functions, has certain advantages over other three models in terms of performance.

**TABLE 8:** The comparison results of different activation functions based models on two data sets.

Data set	Size	MTSL-SCRBN		MTSL-SCSGM		MTSL-SCTANH		MTSL-SCReLU	
		training	test	training	test	training	test	training	test
Stock		0.0796	**0.1105**	0.0819	0.1566	0.0814	0.1586	0.0853	0.1424
	10	(0.0039)	(0.0040)	(0.0048)	(0.0057)	(0.0036)	(0.0036)	(0.0035)	(0.0055)
		0.0661	**0.0904**	0.0766	0.1231	0.0758	0.1242	0.0759	0.1194
	20	(0.0050)	(0.0069)	(0.0032)	(0.0098)	(0.0034)	(0.0076)	(0.0028)	(0.0051)
		0.0542	**0.0793**	0.0671	0.1128	0.0665	0.1037	0.0678	0.1009
	30	(0.0054)	(0.0078)	(0.0020)	(0.0061)	(0.0021)	(0.0076)	(0.0012)	(0.0046)
Sarcos		2.3921	**3.6667**	3.3670	5.1386	3.2588	4.9160	3.1868	5.5491
	700	(0.0221)	(0.0364)	(0.0263)	(0.0868)	(0.0308)	(0.0746)	(0.0140)	(0.0824)
		2.2732	**3.1740**	3.1829	4.0958	3.0747	4.1529	2.9880	4.1854
	1,400	(0.0250)	(0.0179)	(0.0161)	(0.0713)	(0.0178)	(0.0598)	(0.0112)	(0.0767)
		2.1128	**2.9291**	2.9860	3.8698	2.8853	3.8420	2.8539	3.8211
	2,100	(0.0255)	(0.0392)	(0.0136)	(0.0764)	(0.0127)	(0.0494)	(0.0705)	(0.0506)

The results with the minimum test errors are marked in bold.

## 5 Conclusion

In this paper, we propose a multi-task supervised learning framework based on stochastic configuration radial basis network. It can be effectively used in classification and regression problems when a single task has a small number of samples. The series experiment results on the four data sets show the proposed MTSL-SCRBN achieves a good performance compared with some existing methods.

Interesting areas for further directions include using the proposed algorithm in hyperspectral remote sensing image classification and other related research areas, considering the impact of using different activation functions in the network, and trying to explore the range of the sample size of the data set to use the multi-task learning method.

## Data Availability

The original contributions presented in the study are included in the article/Supplementary Material, further inquiries can be directed to the corresponding author.

## Footnotes

1 These four datasets can be obtained from http://archive.ics.uci.edu/ml/datasets/Stock+portfolio+performance, http://gaussianprocess.org/gpml/data, http://bristol.ac.uk/cmm/learning/support/datasets and http://yann.lecun.com/exdb/mnist/, respectively.
