# Corrigendum: Multi-task learning based on Stochastic Configuration Networks

**DOI:** 10.3389/fbioe.2023.1224382

**Published:** 2023-09-01

**Authors:** Xue-Mei Dong, Xudong Kong, Xiaoping Zhang

**Affiliations:** Collaborative Innovation Center of Statistical Data Engineering, Technology and Application, School of Statistics and Mathematics, Zhejiang Gongshang University, Hangzhou, China

**Keywords:** multi-task learning, neural networks, stochastic configuration, knowledge sharing and transfer, supervised mechanism

In the published article, there was an error in the **Article Title**. Instead of “Multi-Task Learning Based on Stochastic Configuration Neural Networks”, it should be “Multi-Task Learning Based on Stochastic Configuration Networks.”

In the published article, there was an error. The authors of “Stochastic Configuration Networks” said that “Stochastic Configuration Networks” is a proper noun. It cannot be written as “Stochastic Configuration Neural Networks.”

A correction has been made to **Abstract**, paragraph 1. This sentence previously stated:

“This paper proposes a multi-task learning framework based on stochastic configuration neural networks. It organically combines the idea of the classical parameter sharing multi-task learning with that of constraint sharing configuration in stochastic configuration neural networks.”

The corrected sentence appears below:

“This paper proposes a multi-task learning framework based on stochastic configuration networks. It organically combines the idea of the classical parameter sharing multi-task learning with that of constraint sharing configuration in stochastic configuration networks.”

The authors apologize for these error and state that this does not change the scientific conclusions of the article in any way. The original article has been updated.

